# Extracellular vesicle neurofilament light is elevated within the first 12-months following traumatic brain injury in a U.S military population

**DOI:** 10.1038/s41598-022-05772-0

**Published:** 2022-03-07

**Authors:** Vivian A. Guedes, Rael T. Lange, Sara M. Lippa, Chen Lai, Kisha Greer, Sara Mithani, Christina Devoto, Katie A. Edwards, Chelsea L. Wagner, Carina A. Martin, Angela E. Driscoll, Megan M. Wright, Kelly C. Gillow, Samantha M. Baschenis, Tracey A. Brickell, Louis M. French, Jessica M. Gill

**Affiliations:** 1 National Institutes of Health, National Institute of Nursing Research, Bethesda, MD 20814 USA; 2 Traumatic Brain Injury Center of Excellence, Silver Spring, MD USA; 3 National Intrepid Center of Excellence, Walter Reed National Military Medical Center, Bethesda, MD USA; 4 General Dynamics Information Technology, Falls Church, VA USA; 5 University of British Columbia, Vancouver, BC Canada; 6 Uniformed Services University of the Health Sciences, Bethesda, MD USA

**Keywords:** Neurodegeneration, Brain injuries, Biomarkers

## Abstract

Traumatic brain injury (TBI) can be associated with long-term neurobehavioral symptoms. Here, we examined levels of neurofilament light chain (NfL) and glial fibrillary acidic protein (GFAP) in extracellular vesicles isolated from blood, and their relationship with TBI severity and neurobehavioral symptom reporting. Participants were 218 service members and veterans who sustained uncomplicated mild TBIs (mTBI, n = 107); complicated mild, moderate, or severe TBIs (smcTBI, n = 66); or Injured controls (IC, orthopedic injury without TBI, n = 45). Within one year after injury, but not after, NfL was higher in the smcTBI group than mTBI (p = 0.001, d = 0.66) and IC (p = 0.001, d = 0.35) groups, which remained after controlling for demographics and injury characteristics. NfL also discriminated the smcTBI group from IC (AUC:77.5%, p < 0.001) and mTBI (AUC:76.1%, p < 0.001) groups. No other group differences were observed for NfL or GFAP at either timepoint. NfL correlated with post-concussion symptoms (r_s_ = − 0.38, p = 0.04) in the mTBI group, and with PTSD symptoms in mTBI (r_s_ = − 0.43, p = 0.021) and smcTBI groups (r_s_ = − 0.40, p = 0.024) within one year after injury, which was not confirmed in regression models. Our results suggest the potential of NfL, a protein previously linked to axonal damage, as a diagnostic biomarker that distinguishes TBI severity within the first year after injury.

## Introduction

Over the last decade, almost 250,000 US service members and veterans (SMVs) have sustained a traumatic brain injury (TBI), with the vast majority classified as mild TBI (mTBI)^1^. Many SMVs who suffered mTBIs recover to premorbid functioning, however, an increasing percentage of SMVs report neurobehavioral symptoms many months or years following injury^2^. Determining blood-based biomarkers that can identify injury severity, and are related to long-term neurobehavioral symptom reporting, may aid in the identification of pathological mechanisms and the development of preventive interventions.

TBI neuropathology consists of a primary injury that results of the traumatic insult, and secondary processes that may lead to glial and neuronal changes, and also increased risk for later-in-life neurodegenerative conditions^3^. Biomarker studies in TBI have focused on circulating levels of proteins associated with pathological processes within the brain, including neurofilament light chain (NfL), a neurofilament protein highly expressed in large-caliber myelinated axons, and glial fibrillary acidic protein (GFAP), a component of the cytoskeleton of astrocytes^4–7^. GFAP is a promising acute TBI biomarker^8^, but a recent study also suggests its informative potential at more chronic timepoints^9^. GFAP levels in serum were suggested to decrease during the first 6 months after TBI, but increase at later timepoints, with elevated levels in patients with moderate or severe TBIs when compared to controls for up to 5 years after injury^9^. NfL has gained increasing attention as a marker of chronic neurodegeneration and poor outcomes in neurological diseases including TBI^10–13^. After TBIs, higher blood NfL has been linked to postconcussive symptoms (PCSx) lasting beyond one year in hockey players^14^, and long-term neurobehavioral symptoms in military populations^15^. In addition, studies have also shown acute and subacute elevations of NfL in moderate and severe TBIs^16,17^. In severe TBI patients, acute NfL levels predicted poor 12-month clinical outcome^18^.

Extracellular vesicles (EVs), such as exosomes, are released by cells throughout the body, including neurons and glia^19,20^. EVs are considered a promising source of biomarkers as they can cross the brain-blood-barrier and their content, which is protected from degradation by a lipid bilayer, reflects the environment of the cell of origin^21–23^. EVs can be isolated from the peripheral circulation, providing an opportunity to investigate brain-specific processes^23,24^. Moreover, levels of proteins within EVs may be related to the biological underpinnings of recovery from a TBI^15,23,25^. EVs play a role in cell signaling and in the removal of unwanted proteins of the brain, and their cargo can functionally change the recipient cells^25,26^. They have also been linked to the pathology of age-related neurodegenerative conditions such as Alzheimer's Disease (AD)^27–29^.

Previous studies have investigated EV levels of NfL^15,30–33^ and GFAP^32,33^ in TBI with variable findings. Our group has reported that EV NfL is chronically elevated in SMVs who sustained repetitive mTBIs (3 or more), and associated with severity of neurobehavioral symptoms^15^. Elevated EV levels of GFAP, but not NfL, have been observed in a civilian population with history of moderate or severe TBI one year post-injury^33^. Importantly, mTBIs are heterogenous according to clinical presentation and neuroimaging findings^34,35^. Uncomplicated and complicated mTBIs are characterized, respectively, by the absence or presence of intracranial abnormalities^34^. To the best of our knowledge, no study has evaluated EV NfL or EV GFAP levels across TBI severities (i.e., mild, moderate, and severe) at distinct chronic timepoints after TBI (i.e., ≤ 1 year, and > 1 year). Similarly, no study has evaluated the potential of EV biomarkers in the stratification of mild injuries in uncomplicated and complicated mTBIs.

In this study, we evaluated SMVs with history of uncomplicated mild, complicated mild, moderate, or severe TBI. We aimed to expand on previous findings and examine whether EV proteins can distinguish TBI patients from controls and TBI severity, as well as their relationship with PCSx and post-traumatic stress disorder (PTSD) symptoms.

## Results

### Demographic and clinical characteristics

Participants (n = 218) were predominately male (94%), white (76%), with a median age of 32 (IQR = 26–42) (Table 1). No significant group differences on demographics were observed, except for time since injury (TSI, months, p = 0.012), which was higher in the mTBI group (median = 35, IQR = 6–92) than in the smcTBI (median = 10.5, IQR = 5–60, p = 0.025) and IC groups (median = 9, IQR = 3–35, p = 0.003). Clinical characteristics, neurobehavioral measures, and biomarker concentrations per TBI severity are provided in Supplemental Table 1 and Supplemental Table 2.

**Table 1:** Demographic and clinical characteristics in IC, mTBI and smcTBI groups.

	IC (n = 45)	mTBI (n = 107)	smcTBI (n = 66)	Significance
**Sex (male)**	41 (91.0%)	100 (93.0%)	63 (95.0%)	X^2^ = 0.85, df = 2, p = 0.655^1^
**Age (years)**				
Median [IQR]	35 [27–43]	32 [26–42]	30 [24–40]	p = 0.599^2^
**Race**				
White	32 (71.0%)	80 (75.0%)	54 (82.0%)	X^2^ = 2.03, df = 4, p = 0.730^1^
African American	6 (13.0%)	13 (12.0%)	5 (7.6%)	
Other	7 (16.0%)	14 (13.0%)	7 (11.0%)	
**Education (years)**				
Median [IQR]	14.00 [13.00–16.00]	14.00 [12.00–16.00]	14.00 [12.00–16.00]	p = 0.510^2^
**TSI (months)**				
Median [IQR]	9 [3–35]	35 [6–92]	10 [5–60]	p = 0.012*^2^
**Number of TBIs**				
Median [IQR]	N/A	1 [1, 2]	1.00 [1–1]	p = 0.050^3^
**TBI severity**^**a**^				
Uncomplicated mTBI	0 (0%)	107 (100%)	0 (0%)	N/A
Complicated mTBI	0 (0.0%)	0 (0.0%)	31 (47.0%)	
Moderate TBI	0 (0.0%)	0 (0.0%)	19 (28.8%)	
Severe TBI	0 (0.0%)	0 (0.0%)	16 (24.2%)	
**PCD Dx (yes)**	12 (30.8%)	30 (32.6%)	19 (32.8%)	X^2^ = 0.07, df = 2, p = 0.965^1^
**NSI total**				
Median [IQR]	19 [12–26]	19.0 [8–31]	15.0 [6–35]	p = 0.857^2^
**PTSD Dx (Yes)**	5 (12.8%)	16 (17.4%)	12 (20.7%)	X^2^ = 1.09, df = 2, p = 0.581^1^
**PCL-C total**				
Median [IQR]	26.0 [21–36]	27.0 [21–37]	24.0 [19–38]	p = 0.509^2^
**Symptom validity**^**b**^				
Yes	39 (89.0%)	92 (86.0%)	58 (89.0%)	X^2^ = 0.46, df = 2, p = 0.796^1^
No	5 (11.1%)	15 (14.0%)	7 (11%)	
Missing	1 (2.2%)	0 (0.0%)	1 (1.5%)	

Statistical tests: ^1^Chi-square test (χ2) test, ^2^Kruskal–Wallis test, ^3^Mann–Whitney U test. *compared between mTBI and smcTBI groups.

IC, injured controls; mTBI, uncomplicated mild TBI; smcTBI, complicated mTBI, moderate TBI, and severe TBI combined; TBI, traumatic brain injury; TSI, time since injury; PCD Dx, classified as meeting DSM-IV Category C symptom criteria for postconcussional disorder; PTSD Dx, classified as meeting DSM-IV-TR symptom criteria for post-traumatic stress disorder; PCL-C, PTSD Checklist-civilian Version; NSI, Neurobehavioral Symptom Inventory.

^a^TBI severity classification: [1] uncomplicated mild TBI: (i) GCS = 13–15, PTA < 24 h, LOC < 30 min, and/or AOC present, and (ii) no trauma-related intracranial abnormality on CT or MRI; [2] complicated mild TBI: (i) GCS = 13–15, PTA < 24 h, LOC < 30 min, and/or AOC present, and (ii) trauma-related intracranial abnormality on CT or MRI; [3] moderate TBI: LOC 30 min–24 h, PTA 1–7 days, and ICA present or absent; and [4] severe TBI: LOC > 24 h, PTA > 7 days, and ICA present or absent.

^b^Participants were not included in the behavioral analysis using NSI and PCLC if they scored below the recommended cutoffs on the validity scales of the MMPI-2-RF.

**Table 2:** Logistic regression analysis comparing groups within one year and one or more years after injury.

	IC versus smcTBI			mTBI versus smcTBI		
	Odds Ratios	SE	p	Odds Ratios	SE	p
**Within one year after injury**						
	**EV NfL**					
AUC	3.78	0.44	**0.002**	2.55	0.33	**0.004**
	82.5% 95% CI 71.59–93.44%			78.2% 95% CI 66.2–90.12%		
	**EV GFAP**					
AUC	3.36	0.64	0.058	1.92	0.52	0.212
	69.9% 95% CI 57.61–82.22%			67.7% 95% CI 56.03–79.45%		
**One or more years after injury**						
	**EV NfL**					
AUC	1.13	0.57	0.828	1.64	0.41	0.225
	70.8% 95% CI 49.47–92.09%			70.4% 95% CI 53.48–87.23%		
	**EV GFAP**					
AUC	0.36	0.80	0.200	1.44	0.59	0.537
	74.4% 95% CI 58.7–90.1%			61.4% 95% CI 47.07–74.93%		

Logistic regression analysis was performed to compare biomarker differences among. Logistic regression models included demographics (age, gender, race) and time since injury when comparing a TBI group and the injured control group. When comparing two TBI groups, number of TBIS was also included in the model. Biomarker concentrations were natural log transformed. P values for significant predictors are marked in bold. Odds ratio, Standard errors (SE) and p values for NfL and GFAP in each model are shown. Area under the Receiver Operating Characteristic Curves (AUCs) for each model are also provided.

IC, injured controls; mTBI, uncomplicated mild TBI; smcTBI, complicated mTBI, moderate TBI, and severe TBI combined; TBI, traumatic brain injury; TSI, time since injury (orthopedic injury or TBI); NfL, Neurofilament light chain; GFAP, glial fibrillary acidic protein.

### NfL discriminates TBI patients within one year after TBI

**Figure 1:**
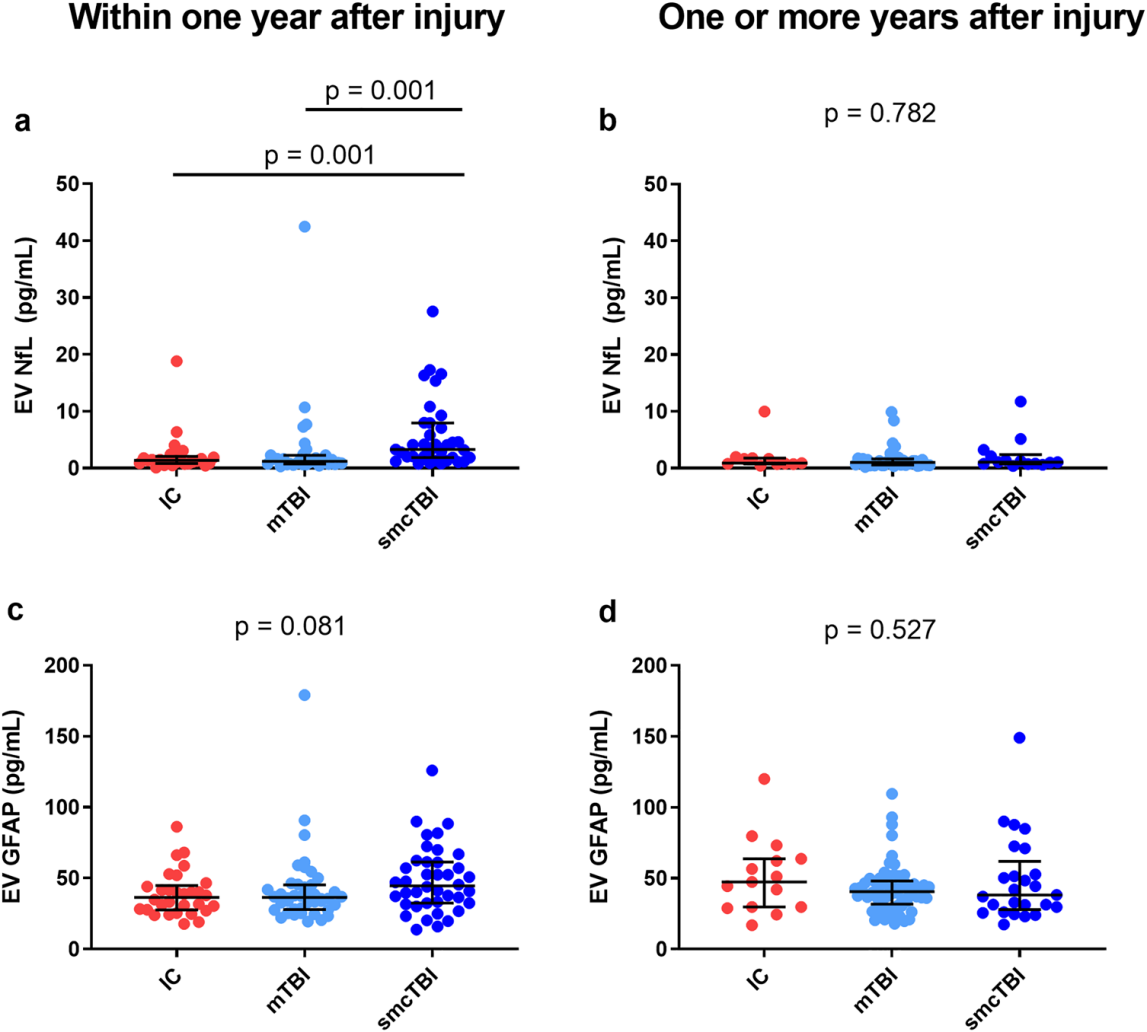
EV levels of biomarkers under one year (**a**,**c**) and one or more years after injury (**b**, **d**). Participants were divided into 3 groups at each timepoint: one or more years after injury (IC, n = 30; mTBI, n = 43; and smcTBI, n = 41) and one more years after injury (IC, n = 15; mTBI, n = 64; and smcTBI, n = 25). Biomarker concentrations are represented as median ± IQR. P values refer to non-parametric pairwise group comparisons adjusted for multiple comparisons ((**a** Dunn’s test correcting for repetitive pairwise comparisons using Bonferroni method) when overall group comparison was statistically significant, or overall group comparison significance (**b**, **c**, **d** Kruskal–Wallis test). IC, injured controls; mTBI, uncomplicated mild TBI; smcTBI, complicated mTBI, moderate TBI, and severe TBI combined; TBI, traumatic brain injury; NfL, neurofilament light chain; GFAP, glial fibrillary acidic protein.

**Figure 2:**
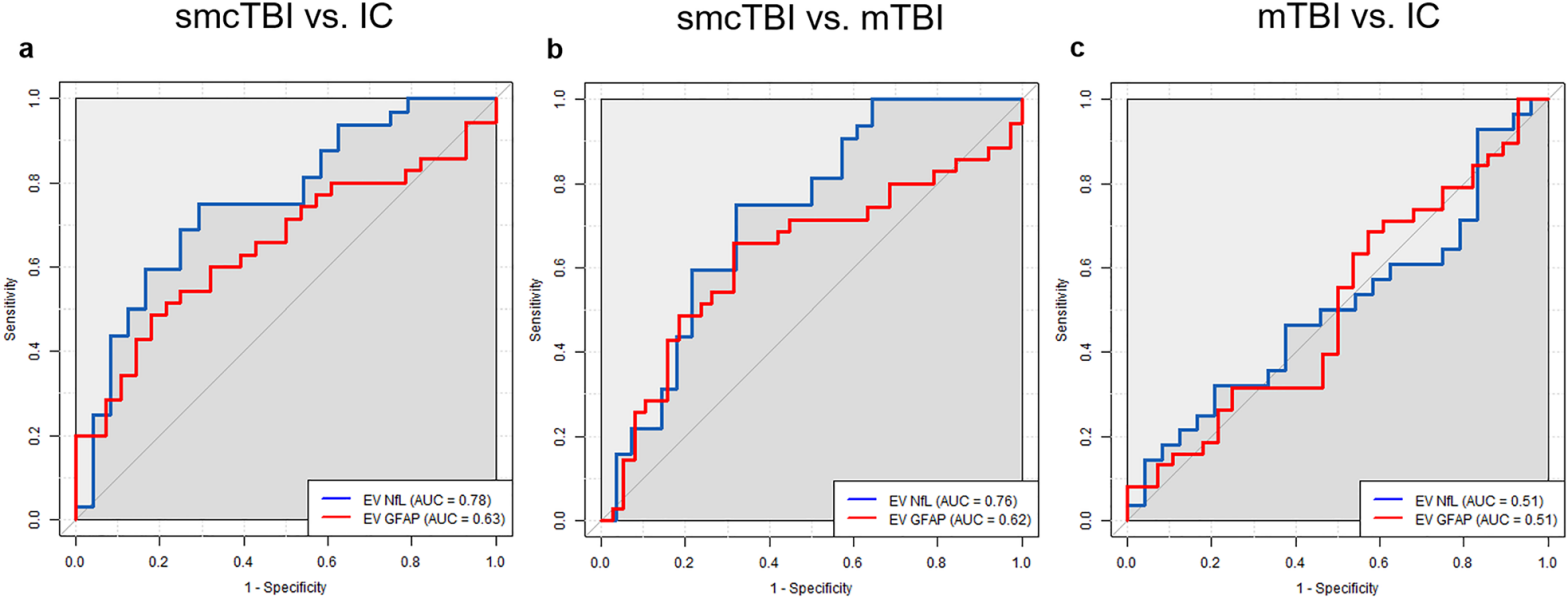
Univariate area under the receiver operating characteristic curves (AUCs) for EV NfL (blue) and EV GFAP (red) within one year after injury comparing smcTBI versus IC (**a**), smcTBI versus mTBI (**b**), and mTBI versus IC (**c**). Grey diagonal line represents reference line (AUC = 0.5). TSI, time since injury; GFAP, Glial fibrillary acidic protein; NfL, Neurofilament light chain; IC, injured controls; mTBI, uncomplicated mild TBI; smcTBI, complicated mTBI, moderate TBI, and severe TBI combined.

Participants were divided according to their TSI (months). Within one year after injury (n = 114, median = 5 months, IQR = 3–8), there were significant group differences for EV NfL (p < 0.001), but not EV GFAP (p = 0.081) when comparing smcTBI (n = 41), mTBI (n = 43), and IC (n = 30) groups (Fig. 1). EV NfL levels were higher in the smcTBI group when compared to IC (p = 0.001, d = 0.66) and mTBI (p = 0.001, d = 0.35) groups, which was confirmed in logistic regression models controlling for demographics and TSI (Table 2). Univariate under the receiver-operating characteristic curve (AUC) analysis revealed that NfL distinguished smcTBI group from IC (AUC = 77.5%, 95% CI 65.3–89.7%, p < 0.001) and mTBI groups (AUC = 76.1%, 95% CI 64.2–88.0%, p < 0.001), but not mTBI from the IC group (AUC = 50.84%, 95% CI 35.31–66.36%, p = 0.546) (Fig. 2). Similarly, EV GFAP distinguished smcTBI group from IC (AUC = 63.08%, 95% CI 49.89–76.27% p = 0.031) and mTBI groups (AUC = 62.14%, 95% CI 49.59–74.69%, p = 0.029), but not mTBI and IC groups (AUC = 50.79%, 95% CI 36.87–64.72%, p = 0.457). AUC values for logistic regression models comparing TBI groups were also calculated (Table 2). One or more years after injury (n = 104, median = 61, IQR = 36–120), we found no significant differences in biomarker levels when comparing smcTBI (n = 25), mTBI (n = 64), and IC (n = 15) groups, for EV NfL (p = 0.659) and GFAP (p = 0.567) (Fig. 1). Logistic regression models also confirmed these findings (Table 2). Neither EV NfL nor EV GFAP discriminated smcTBI from IC (AUC = 53.9%, 95% CI 29.78–78.01% for NfL; AUC = 54.4%, 95% CI 35.19–73.61%, for GFAP) or mTBI groups (AUC = 44.82%, 95% CI 27.41–62.23%, for NfL; AUC = 48.81%; 95% CI 33.71–63.91%, for GFAP) one or more years after the injury. No statistically significant differences in biomarker levels between IC and mTBI groups were observed at either timepoint. In addition, neither GFAP nor NfL distinguished mTBI and IC groups. Biomarker levels across TBI severity at each timepoint are also reported in Supplemental Table 3 and Supplemental Table 4.

### TBI characteristics and biomarker levels

**Figure 3:**
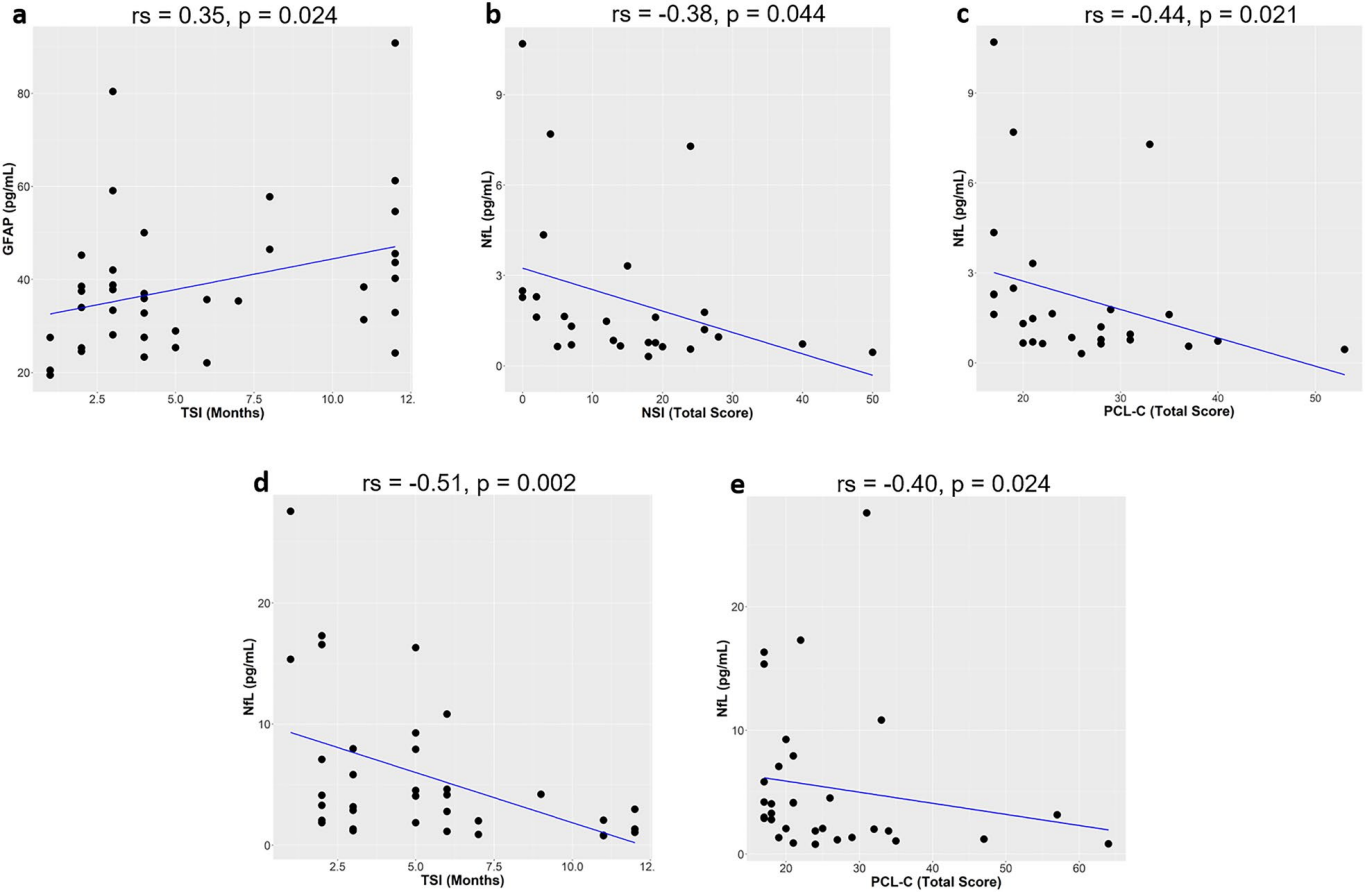
Correlations between biomarker levels and time since injury (TSI, months) or behavioral scores within one year of injury in the mTBI group (**a**–**c**) and smcTBI group (**d**,**e**). For each correlation, rho (r_s_) and p values are provided (Spearman rank correlations). Regression line is shown in blue. One influential data point was removed from the mTBI group as it greatly affected the slope of the regression line involving either NfL or GFAP. Concentrations of NfL and GFAP for the data point were higher than the median plus 3 median absolute deviations. GFAP, glial fibrillary acidic protein; NfL, neurofilament light chain; TSI, time since injury (months); PCL-C, PTSD Checklist-civilian Version; NSI, Neurobehavioral Symptom Inventory.

We analyzed correlations between biomarker levels and TSI (months). Within one year after TBI, the median TSI was 4.0 (IQR = 3.0–8.0) in the mTBI group and 5.0 in the smcTBI group (IQR = 3.0–8.0). EV GFAP levels positively correlated with TSI (r_s_ = 0.35 , p = 0.024) in the mTBI group, while NfL levels negatively correlated with TSI in the smcTBI group (r_s_ = − 0.51, p = 0.002). Concentrations of EV NfL and EV GFAP in relation to TSI are graphically represented in Fig. 3. One or more years after injury, the median TSI was 66.5 (IQR = 36–120) in the mTBI group and 118 (IQR = 60–120) in the smcTBI group, but the difference was not statistically significant (p = 0.149). No significant correlations between TSI and biomarker levels were observed. In the mTBI group, the correlation between EV NfL levels and number of TBIs was marginally significant (r_s_ = 0.29, p = 0.064).

### Biomarker levels and behavioral symptoms

Within one year after injury, EV NfL levels correlated with NSI (r_s_ = − 0.38, p = 0.044) in the mTBI group but not in the smcTBI group (r_s_ = − 0.32, p = 0.074) (Fig. 3). EV NfL levels also correlated with PCL-C in the mTBI (r_s_ = − 0.44, p = 0.021) and smcTBI groups (r_s_ = − 0.40, p = 0.024). However, associations between EV NfL levels and symptoms were not confirmed in regression models including demographics, TSI, and number of lifetime TBIs. No other associations between biomarkers and NSI or PCL-C scores were observed.

## Discussion

Our main finding suggests a dose–response relationship between TBI severity and concentrations of NfL in EVs within one year after TBI. Specifically, participants who had sustained a complicated mTBI, moderate or severe TBI had higher levels of blood EV NfL than those with uncomplicated mTBI or injured controls. Additionally, correlation analysis suggests an increase with time in levels of EV GFAP in participants with uncomplicated mTBI, and a decrease in EV NfL in more severe injuries, within the first year after TBI. Our findings support the potential of EV NfL as a diagnostic biomarker in TBI and as a means to discriminate TBI severity, shedding light on underlying pathology mechanisms.

NfL is a component of the neuronal cytoskeleton and a marker of axonal injury and degeneration^36,37^. Several recent studies have indicated the prognostic and predictive value of measuring concentrations of NfL in the peripheral blood in chronic neurological diseases including amyotrophic lateral sclerosis (ALS)^38^, multiple sclerosis (MS)^39^, AD^40^, Parkinson’s Disease (PD)^13^, as well as peripheral neuropathies^41^. In PD patients, NfL has shown potential as a predictive biomarker of disease severity and progression based on motor and cognitive measures^13,42^. Higher plasma NfL has also been linked to higher risks of developing dementia in a non-demented population^40^. In TBI, chronically elevated levels of NfL in individuals with multiple mTBIs have also been shown, in association with more severe neurobehavioral symptoms in athletes and military populations^14,15,36,43–45^. Other studies have observed early increases in the NfL levels after moderate and severe TBIs^16,17^. A recent study has shown that serum NfL levels decreased in a linear fashion over the first 5-years after mild to moderate TBI^9^. NfL distinguished participants with moderate to severe TBI from controls with higher AUCs at 30, 90, and 180 day time points. NfL also distinguished mTBI from controls at the 30-day time point^9^. Nevertheless, the temporal profile of NfL release in the blood after TBIs is not completely understood. GFAP is an important component of the cytoskeleton of astrocytes and has been considered a biomarker of astroglial injury^46^. Changes in astrocyte function have been associated with aging and neurological diseases^47,48^. In TBI, several studies have provide strong indications of the value of GFAP as an acute biomarker, but links between changes in astrocyte function and TBI pathology are only beginning to be understood^4,5,49–52^.

EVs have a complex biogenesis that is yet to be completely understood, and are heterogenous in size, content and origin^23,53^. They play a role in local and systemic intercellular signaling^54–56^. In addition, EVs have been linked to the pathology of age-related neurodegenerative conditions such as AD with a role in packaging and spread of misfolded proteins^27,28,57,58^. TBI pathology shares similarities with progressive neurological conditions, and is also associated with neurogenerative processes, suggesting the potential of EV NfL as a biomarker in chronic phases after the TBI.

Our group has previously reported higher EV NfL in SMVs with repetitive mTBI in comparison to those with 1 or 2 TBIs in a cohort in average 8 years after the last injury, with a positive correlation between NfL and TSI^15^. In the same study, we also found links between higher levels of EV NfL and more severe PCSx and PTSD symptoms. Here, correlations between the severity of symptoms and biomarker levels were observed within the first year of injury, but these findings were not confirmed in regression models. We observed no significant correlations between NfL, PCSx and PTSD symptoms after one year following the injury in the mTBI group as we have previously reported. Moreover, correlations between number of TBIs and concentrations of EV NfL in the mTBI group were only marginally significant. These differences between studies might be due to a higher number of participants with repetitive mTBI and lifetime mTBIs in the previous study, where participants could have up to 6 lifetime TBIs, compared to a maximum number of 3 in the present study. We hypothesize that a single mTBI may not induce severe enough injury to the brain to result in chronic elevation of peripheral levels of NfL, and that higher blood NfL in those with repetitive mTBI and in the smcTBI group might reflect more severe axonal degeneration.

Biological relevance and time course of EV release after TBI are still unclear, but they might depend at least partially on the mechanism of injury^32^. Our group has previously reported that moderate-to-severe TBI patients with diffuse injury displayed higher levels of EV NFL and EV GFAP in serum than those with focal lesions at acute timepoints after injury^32^. Moreover, EVs play a role in maintenance of central nervous system homeostasis and in the removal of unwanted proteins of the brain^59–61^, and it is plausible that EVs function as a mechanism to remove products of TBI-induced damage and pathological processes from the brain.

In this study, within one year after injury, EV NfL was significantly higher in participants with more severe injuries when compared to those with uncomplicated mTBI and controls. Median levels of GFAP were also higher in more severe injuries, but group differences were only marginally significant. EV NfL also discriminated those in the smcTBI group from mTBI and IC groups, especially when controlling for demographics and injury characteristics, outperforming GFAP. No group differences in either NfL or GFAP were observed after one year of injury. Interestingly, a recent study has shown significantly higher levels of EV GFAP in moderate and severe TBIs when compared to controls, but not EV NfL, in a civilian cohort at one year after the injury^33^. It is possible that these discrepancies are due in part to differences in the relative number of severe TBI cases and to a decrease in blood concentrations of EV NfL within the first year after the TBI. Accordingly, we observed in this study a negative correlation between concentration of EV NfL and time in months within the first year after injury. Here, we also observed an increase in the levels of EV GFAP with time in the mTBI groups, which could be an indication of the development of chronic pathological processes involving astrocytes. Importantly, we had a smaller sample size in the group with one or more years since the injury, with a lower percentage of participants with more severe injuries, than in the group within one year after injury. These differences between groups could be a potential confounder, and should be considered when interpreting our results. Future studies with larger samples sizes are warranted to confirm our findings and to further evaluate the prognostic potential of EV NfL and EV GFAP at distinct timepoints after injury across TBI severities. Moreover, temporal profiles of EV biomarkers should be further investigated, addressing a possible biphasic release of EV NfL and EV GFAP, with a decrease within the first year after injury followed by increased levels at more chronic timepoints.

Strengths of this study include the rigorous categorization of TBIs and symptom validation by using the MMPI-2-RF. Nevertheless, this study has limitations, which include the use of cross-sectional data, variability in time since injury at each timepoint, as well as self-reported behavioral symptoms. In addition, the relatively small sample size reduced the sensitivity of our analysis. The smaller number of participants with one or more years after TBI than within the first year after TBI, especially in the group with more severe injury, might be a confounding factor in this study. Moreover, our cohort was predominantly white and male, which reduces the generalizability of our results to other populations. Sex may be linked to differences in the biological response to injuries as well as brain repair processes and recovery^62–65^. Additionally, sociocultural factors in association with conspicuous disparities in healthcare access and utilization may influence recovery and clinical outcomes after TBI in more racially/ethnically diverse cohorts^66–68^. Future efforts are warranted to further explore the biomarker utility of EV proteins as biomarkers in TBI in more representative samples, as well as effects of sex and sociocultural differences in TBI pathology and recovery. Moreover, NfL cannot discriminate neuro-axonal injury of the central and peripheral nervous system. Thus, NfL levels might also reflect peripheral nerve damage caused by body injury. Our findings support the potential of EV NfL as a diagnostic biomarker in TBI and as a means to discriminate TBI severity. Increased levels of NfL in TBI ranging from complicated mild to severe suggest that persistent axonal degeneration or remodeling occur in the first year after the TBI. Additional studies are warranted to validate our results, examining longitudinal changes in EV NfL and EV GFAP.

## Methods

### Study design and population

Participants were 215 SMVs enrolled in the 15‐Year Longitudinal TBI Study (Defense and Veterans Brain Injury Center [DVBIC]/Traumatic Brain Injury Center of Excellence[TBICoE]), recruited from inpatient and outpatient clinics at the Walter Reed National Military Medical Center (WRNMMC; Bethesda, MD) from 2011 to 2019. Participants were active-duty service members or other Defense Enrollment Eligibility Reporting System-eligible veteran; and 18 years of age or older who could read and understand English. Exclusion criteria included: a lack of proficiency in conversational English, or a history of significant neurological or psychiatric condition(s) unrelated to the injury event or deployment (e.g., Meningioma, Bipolar Disorder). Determination of TBI diagnosis and severity was based on medical records and an interview as previously described^2,69^. Participants were divided into three injury groups: Injury Controls (IC, orthopedic injury without TBI, n = 45), mTBI (uncomplicated TBIs only, n = 107) and smcTBI (complicated mTBI, moderate, or severe TBIs, n = 66). All participants provided written informed consent to participate. This study was approved by the WRNMMC Institutional Review Board in Bethesda, MD and was conducted in accordance with the guidelines of the Declaration of Helsinki.

### Determination of TBI severity and orthopedic injury

TBI severity was classified as follows: [1] uncomplicated mild TBI: (i) Glasgow Coma Scale score (GCS) = 13–15, post-traumatic amnesia (PTA) < 24 h, loss of consciousness (LOC < 30 min), and/or alteration of consciousness/mental state (AOC) present, and (ii) no trauma-related intracranial abnormality (ICA) on computed tomography (CT) or structural magnetic resonance imaging (MRI); [2] complicated mild TBI: (i) GCS = 13–15, PTA < 24 h, LOC < 30 min, and/or AOC present, and (ii) trauma-related ICA on CT or MRI; [3] moderate TBI: LOC 30 min–24 h, PTA 1–7 days, and ICA present or absent; and [4] severe TBI: LOC > 24 h, PTA > 7 days, and ICA present or absent. Participants were classified into the Injured Control group if the following was observed: (i) they experienced an orthopedic injury event, (ii) no evidence of an altered state of consciousness as a result of the injury (e.g., GCS < 15; AOC, LOC, and PTA present) or ICA, and (iii) no history of TBI. Neuroimage evaluation was performed at the National Intrepid Center of Excellence (NICoE), which is located on the campus of Walter Reed National Military Medical Center (WRNMMC). All participants received a CT or MRI to evaluate possible of intracranial abnormalities as part of a two-day evaluation that also included clinical interviews and a variety of neurobehavioral measures.

### Assessment of PCSx and PTSD symptoms

Participants completed a 2.5‐hour battery of self‐report neurobehavioral measures. PTSD Checklist–Civilian version (PCL-C)^70^ and Neurobehavioral Symptom Inventory (NSI)^71^ were used to evaluate PTSD symptoms and PCSx, respectively. Higher scores in PCL-C and NSI indicate more severe symptoms. Participants’ responses on the PCL-C were used to create two PTSD categories based on DSM-IV-TR symptom criteria for PTSD. A participant was classified into the PTSD-Present category based on the endorsement of moderate or higher symptoms for (a) one or more Criterion B symptoms, (b) three or more Criterion C symptoms, and (c) two or more Criterion D symptoms. Similarly, participants’ responses on the NSI were also used to classify DSM-IV-TR research criteria for Postconcussional Disorder (PCD). A participant was classified into the PCD-Present category based on the endorsement of moderate or higher symptoms for (a) three or more Category C symptoms, and (b) subjective complaints of attention or memory problems (i.e., Category B; note that Category B criteria require objective evidence of cognitive impairment in attention or memory. For the purposes of this study, subjective reports of these cognitive complaints were used as a proxy). Participants also completed the Minnesota Multiphasic Personality Inventory-2, Restructured Form (MMPI-2-RF)^72^ to evaluate symptom validity and were not included in the behavioral analysis if they scored above the recommended cutoffs on the validity scales (i.e., Infrequent Responses [F-r] ≥ 100 T or Infrequent Psychopathology Responses [Fp-r] ≥ 90 T or Infrequent Somatic Responses [Fs] ≥ 100 T or Symptom Validity [FBS-r] ≥ 100 T or Response Bias Scale [RBS43] ≥ 100 T). The number of participants who scored above the cutoff on MMPI-2-RF in the IC, mTBI, and smcTBI groups were 5 (11%), 15 (14%), and 7 (11%), respectively. Additionally, the validity data was missing for one participant in the IC group, and one participant in the smcTBI group.

### Blood sampling and EV isolation from serum

Non-fasting blood samples were collected using serum separator tubes (SST) and processed using standard protocols^73^. Serum was aliquoted, stored at − 80 °C until EV isolation. EVs were isolated from 0.8 ml of frozen serum. After thawing the serum, samples were centrifuged at 3000 × g for 15 min at 4 °C to remove debris and the supernatant was transferred into a clean tube. ExoQuick solution (System Biosciences Inc., Mountainview, CA) was added to the samples according to the manufacturer instructions. Samples were incubated for 1 h at 4 °C and centrifuged at 1500 g for 30 min. After the centrifugation, the supernatant was aspirated and the pellet was resuspended in 0.8 ml of Dulbecco’s calcium- and magnesium free salt solution (Sigma-Aldrich, St. Louis, MO, USA). Resuspended pellets containing EVs were stored at − 80 °C and later used to measure total EV protein content and particle characterization. For particle characterization, samples were analyzed using MACSPlex Exosome Kit (130-108-813, Miltenyi Biotec, Bergisch Gladbach, Germany) and nanoparticle tracking analysis (NTA) software (Malvern Instruments, Malvern, United Kingdom) to determine the mean diameter (nm) and concentration (particles/mL) of EVs.

### Protein quantification

For each participant, 100 µl of the EV sample were used to measure total protein content. Each sample received the same volume of M-PER mammalian protein extraction reagent to lyse EV (Thermo Scientific, Inc., Rockford, IL), containing three times the suggested concentrations of protease and phosphatase inhibitors (EASYpack Protease Inhibitor Cocktail, catalog number 5892791001, Millipore Sigma). These mixtures were analyzed using a site-specific Simoa HD-1 analyzer (Quanterix, Lexington, MA). EV concentrations NfL, GFAP, Tau and Ubiquitin C-Terminal Hydrolase L1 (UCHL1) were measured using an ultrasensitive paramagnetic bead-based enzyme-linked immunosorbent assay (Neurology 4-plex A, item 102,153, Quanterix), according to the manufacturer’s protocol. Samples were randomized over plates, run in duplicate, with laboratory scientists blinded to participant groups. The accepted coefficients of variation (CVs) of analyzed samples were no higher than 30% for all analytes. Results for UCHL1 and tau did not meet our quality standards as a large percentage of samples (71.6% and 77.5% for tau and UCHL1, respectively) had non-detectable levels of protein or CVs higher than 30%. Thus, we only report here results for NfL and GFAP. Average CVs were 11% (SD = 7.5) and 3% (SD = 2.5%) for NfL and GFAP, respectively.

### Statistical analysis

Comparison of demographic and clinical characteristics between groups were conducted using Chi-square test (χ2), Mann–Whitney U tests or Kruskal–Wallis test. Biomarker comparisons among TBI groups were performed by using Kruskal–Wallis test followed by Dunn’s test correcting for repetitive pairwise comparisons using Bonferroni method. In addition, we performed logistic regression analysis, and calculated the AUC for each model. Univariate AUCs for each biomarker were also calculated. Logistic regression models included demographics (age, gender, race) and time since injury when comparing a TBI group and the injured control group. When comparing two TBI groups, number of TBIs was also included in the model. Confidence intervals were calculated by using DeLong’s method. Cohen’s d was calculated for biomarker pairwise group comparison. Spearman correlations and regressions analysis (negative binomial generalized linear models, GLMs) were used to examine relationships between biomarkers and PCSx and PTSD symptoms. The GLMs included age, gender, race, time since injury, and number of TBIs. Outcomes were either PCL-C or NSI total scores. All data were analyzed using R version 4.0.2. R and GraphPad Prism version 7.04 were used to produce graphs.

## Supplementary Information

Supplementary Information.

## Data Availability

Aggregate data that support the findings of this study are available upon reasonable request to the corresponding author.
