# Supplementary material for: Extracellular vesicle neurofilament light is elevated within the first 12-months following traumatic brain injury in a U.S military population
Source: Sci Rep. 2022 Mar 7;12:4002. doi: 10.1038/s41598-022-05772-0 (PMC8901614; doi:10.1038/s41598-022-05772-0)
Supplement: Supplementary file 1 — Supplementary Information. [file 41598_2022_5772_MOESM1_ESM.docx]

**Supplemental Table 1:** TBI characteristics and behavior scores for each TBI severity within one year after injury

| **Characteristic** | **IC**  N = 30*^1^* | **Uncomplicated**  **mTBI**  N = 43*^1^* | **Complicated**  **mTBI**  N = 18*^1^* | **Moderate/Severe**  **TBI**  N = 23*^1^* | **p-value***^2^* |
| --- | --- | --- | --- | --- | --- |
| TSI | 6.5 (3.0- 8.8) | 4.0 (3.0- 8.0) | 3.0 (2.2- 6.8) | 6.0 (5.0- 10.5) | 0.5 |
| Number of TBIs |  |  |  |  |  |
| 0 | 30 (100%) | 0 (0%) | 0 (0%) | 0 (0%) |  |
| 1 | 0 (0%) | 35 (81%) | 17 (94%) | 22 (96%) |  |
| 2 | 0 (0%) | 6 (14%) | 1 (5.6%) | 1 (4.3%) |  |
| 3 | 0 (0%) | 2 (4.7%) | 0 (0%) | 0 (0%) |  |
| NSI | 18 (13- 24) | 13 (4- 22) | 12 (6- 23) | 13 (4- 22) | 0.5 |
| PCL-C | 27 (21- 35) | 22 (19- 30) | 21 (19- 26) | 22 (18- 30) | 0.4 |
| Symptom Validity (yes) | 28  (97%) | 39 (91%) | 14 (78%) | 22 (100%) | 0.7 |

Footnotes: Participants were not included in the behavioral analysis using NSI and PCLC if they scored below the recommended cutoffs on the validity scales of the MMPI-2-RF. ^1^Statistics presented: Median (IQR); n (%). ^2^Statistical tests performed: Kruskal-Wallis test. Abbreviations: IC, injured controls; TBI, traumatic brain injury; mTBI, mild traumatic brain injury; TSI, time since injury; PCL-C, PTSD Checklist-civilian Version; NSI, Neurobehavioral Symptom Inventory.

**Supplemental Table 2:** TBI characteristics and behavior scores for each TBI severity one or more years after injury

| **Characteristic** | **IC**  N = 15*^1^* | **Uncomplicated**  **mTBI**  N = 64*^1^* | **Complicated**  **mTBI**  N = 13*^1^* | **Moderate/Severe**  **TBI**  N = 12*^1^* | **p-value^2^** | |
| --- | --- | --- | --- | --- | --- | --- |
| TSI | 55  (35- 87) | 66  (36- 120) | 120  (60- 120) | 118  (54- 120) | 0.1486 | |
| Number of TBIs |  |  |  |  |  | |
| 0 | 15 (100%) | 0 (0%) | 0 (0%) | 0 (0%) |  | |
| 1 | 0 (0%) | 43 (67%) | 8 (62%) | 9 (75%) |  | |
| 2 | 0 (0%) | 13 (20%) | 5 (38%) | 2 (17%) |  | |
| 3 | 0 (0%) | 8 (12%) | 0 (0%) | 1 (8.3%) |  | |
| NSI | 23  (10- 32) | 24  (11- 35) | 40  (18- 49) | 12  (7- 32) | 0.2 | |
| PCL-C | 24  (22-40) | 32  (22-43) | 48  (27-56) | 24  (19-38) | 0.4 |  |
| Symptom Validity (yes) | 11 (73%) | 53 (83%) | 12 (92%) | 10 (83%) |  |  |

Footnotes: Participants were not included in the behavioral analysis using NSI and PCLC if they scored below the recommended cutoffs on the validity scales of the MMPI-2-RF. ^1^Statistics presented: Median (IQR); n (%). ^2^Statistical tests performed: Kruskal-Wallis test. Abbreviations: IC, injured controls; TBI, traumatic brain injury; mTBI, mild traumatic brain injury; TSI, time since injury; PCL-C, PTSD Checklist-civilian Version; NSI, Neurobehavioral Symptom Inventory.

**Supplemental Table 3:** Biomarker levels for each TBI severity within one year after injury

|  | **IC**  N = 30 | **Uncomplicated**  **mTBI**  N = 43 | | **Complicated**  **mTBI**  N = 18 | **Moderate/**  **Severe**  **TBI**  N = 23 | **p-value** | |
| --- | --- | --- | --- | --- | --- | --- | --- |
| **EV NFL (pg/mL)** |  |  | |  |  |  | |
| Median (IQR) | 1.35  (0.8-1.9) | 1.20  (0.7-2.3) | | 2.78  (1.3-5.8) | 4.11  (2.0-8.9) | **<0.001** | |
| Mean (SD) | 2.28 (3.69) | 3.36 (7.64) | | 4.31 (4.36) | 6.60 (6.92) |  | |
| EMM  (95% CI) | 2.29 (-0.18, 4.83) | 3.36 (1.00, 5.50) | | 4.30 (0.88, 7.67) | 6.60 (3.88, 9.07) |  | |
| **EV GFAP (pg/mL)** |  |  | |  |  |  | |
| Median (IQR) | 36.39  (28-44) | 36.41  (28-45) | | 40.01  (30-48) | 52.47  (37-66) | 0.058 | |
| Mean (SD) | 38.85 (15.57) | 41.96 (26.25) | | 41.45 (17.74) | 53.92 (25.77) |  | |
| EMM  (95% CI) | 38.91 (31.10, 47.38) | 41.95 (35.28, 48.63) | | 41.32 (30.77, 52.21) | 53.80 (44.85, 63.09) |  | |
|  |  |  |  | |  | |  |

Footnotes: Significant group differences were observed for NfL, but no GFAP. P values refer to group comparisons (Kruskal-Wallis test) for NfL (p<0.001) or GFAP levels (p=0.0578). Pairwise comparison using Dunn's test with Bonferroni correction for multiple comparisons revealed that EV NfL levels were higher in the Moderate/Severe TBI group when compared to IC (p = 0.003, d = 0.79) and Uncomplicated mTBI (p = 0.002, d = 0.44), but not complicated mTBI (p = 1.000, d = 0.37). Abbreviations: IC, injured controls; TBI, traumatic brain injury; mTBI, mild traumatic brain injury; EMM, Estimated Marginal Mean; CI, confidence interval; NfL, neurofilament light chain; GFAP, glial fibrillary acidic protein.

**Supplemental Table 4:** Biomarker levels for each TBI severity one or more years after injury

|  | **IC**  N = 15 | **Uncomplicated**  **mTBI**  N = 64 | **Complicated**  **mTBI**  N = 13 | **Moderate/**  **Severe**  **TBI**  N = 12 | **p-value** | |
| --- | --- | --- | --- | --- | --- | --- |
| **EV NFL (pg/mL)** |  |  |  |  |  | |
| Median (IQR) | 0.88  (0.75-1.71) | 1.19  (0.57-1.66) | 1.00  (0.82-1.31) | 1.16  (0.79-2.40) | 0.914 | |
| Mean (SD) | 1.94 (2.71) | 1.71 (2.03) | 2.75 (4.41) | 1.85 (1.62) |  | |
| EMM  (95% CI) | 1.93 (0.56, 3.47) | 1.71 (0.99, 2.50) | 2.72 (0.74, 4.56) | 1.84 (0.16, 3.57) |  | |
| **EV GFAP (pg/mL)** |  |  |  |  |  | |
| Median (IQR) | 47.41  (30-63) | 40.29  (32-47) | 32.86  (31-50) | 43.29  (26-58) | 0.669 | |
| Mean (SD) | 51.36 (26.5) | 42.29 (17.0) | 44.55 (22.8) | 53.21 (36.6) |  | |
| EMM  (95% CI) | 51.59 (41.42, 63.25) | 42.24 (37.38, 47.79) | 44.54 (32.25, 56.97) | 53.12 (41.43, 66.36) |  | |
|  |  |  |  |  | |  |

Footnotes: No statically significant differences on biomarker levels among groups was observed. P values refer to group comparisons (Kruskal-Wallis test) for NfL (p = 0.9142) or GFAP (p=0.6686). Abbreviations: IC, injured controls; TBI, traumatic brain injury; mTBI, mild traumatic brain injury; TSI, time since injury; PCL-C, PTSD Checklist-civilian Version; NSI, Neurobehavioral Symptom Inventory; EMM, Estimated Marginal Mean. Abbreviations: IC, injured controls; TBI, traumatic brain injury; mTBI, mild traumatic brain injury; EMM, Estimated Marginal Mean; CI, confidence interval; NfL, neurofilament light chain; GFAP, glial fibrillary acidic protein.
